# Involvement of IL-10 and TGF-β in HLA-E-mediated neuroblastoma migration and invasion

**DOI:** 10.18632/oncotarget.10041

**Published:** 2016-06-14

**Authors:** Zijun Zhen, Xiaofang Guo, Ru Liao, Kaibin Yang, Litong Ye, Zhiyao You

**Affiliations:** ^1^ Department of Pediatric Oncology, Sun Yat-sen University Cancer Center, Guangzhou, 510060, China; ^2^ State Key Laboratory of Oncology in South China, Guangzhou, 510060, China; ^3^ Collaborative Innovation Center of Cancer Medicine, Guangzhou, 510060, China; ^4^ Zhongshan School of Medicine, Sun Yat-Sen University, Guangzhou, 510080, China

**Keywords:** neuroblastoma, migration, invasion, HLA-E, IL-10

## Abstract

Human leukocyte antigen (HLA)-E is highly expressed in a variety of tumors and, in addition to immune escape, may promote tumor growth via other mechanisms. However, the role of HLA-E in neuroblastoma (NB) migration and invasion is unknown. In the present study, HLA-E expression in human NB tumors was measured by immunohistochemistry. The effect of HLA-E on NB cell migration and invasion was studied *in vitro* and *in vivo*, as well as the effect of HLA-E on natural killer (NK)-cell cytotoxicity. HLA-E was expressed in 70.2% of the NB tumor tissues examined. HLA-E expression by NB cells inhibited NK-cell cytotoxicity and induced the release of interleukin (IL)-10 and transforming growth factor (TGF)-β1. HLA-E and the released cytokines enhanced the ability of NB cells migration and invasion. NK cell infusion did not inhibit the growth of NB cells with high HLA-E expression but instead increased the number of metastatic cells in the bone marrow. Taken together, the results indicate that IL-10 and TGF-β are involved in HLA-E-mediated NB migration and invasion. Thus, HLA-E may be a new treatment target in NB.

## INTRODUCTION

Neuroblastoma (NB), a high-grade malignancy with poor prognosis, originates from the primitive neural crest and is the most common extra-cranial solid tumor in children. More than 70% of NB patients have metastatic disease at diagnosis, which is classified as high- risk NB. Though current multidisciplinary treatment is available, the 5-year progression-free survival of high-risk NB patients is only 36%–56% [1–2]. Therefore, new therapies are needed to improve treatment outcomes in NB patients. The early metastasis suggests that NB cells are highly competent. Gaining a thorough understanding of the regulatory mechanism underlying NB migration and invasion would help identify new therapeutic targets.

Classic human leukocyte antigen-I (HLA-Ia) molecules are reduced or absent on the surface of tumor cells, whereas non-classic HLA (HLA-Ib) molecules, such as HLA-G and HLA-E, are highly expressed on the surface of tumor cells. HLA-E is the main ligand for the inhibitory receptor CD94/NKG2A on the surface of natural killer (NK) and cytotoxic T lymphocyte (CTL) cells. In the major mechanism underlying tumor immune escape, HLA-E binds to the leading peptides of HLA-A, -B, -C, and -G, activating the CD94/NKG2A receptor and delivering an inhibitory signal to NK and CTL cells [3]. Studies have found that HLA-E is highly expressed in a variety of tumors [4] and closely associated with certain biological characteristics of tumors [5]. However, HLA-E expression in NB tissue and its clinical significance is yet to be determined.

In addition to immune escape, HLA-E may promote tumor growth via other mechanisms related to tumor stem cell differentiation [6], tumor-associated macrophage activity [7], and functional activation of the vascular endothelium [8]. In a cohort of laryngeal carcinoma patients, HLA-E may be a biomarker of tumor invasiveness [9], but the definitive mechanism is unknown. Hepatic cells infected with hepatitis virus have been shown to express a high level of HLA-E, which inhibits NK-cell cytotoxicity in abnormal hepatic cells and stimulates NK cells to release cytokines, including interleukin (IL)-10 and transforming growth factor (TGF)-β1 [10]. In breast cancer and lung adenocarcinoma, IL-10 and TGF-β1 have been confirmed to promote tumor growth through pathways including tumor migration and invasion [11–12]. NB is an aggressive tumor. Therefore, we hypothesize that HLA-E is highly expressed in NB tissue, inhibits NK cells, and promotes the release of IL-10 and TGF-β1 to affect tumor growth.

## RESULTS

### Expression of HLA-E in NB was correlated to disease stage and N-MYC gene status

**Figure 1:**
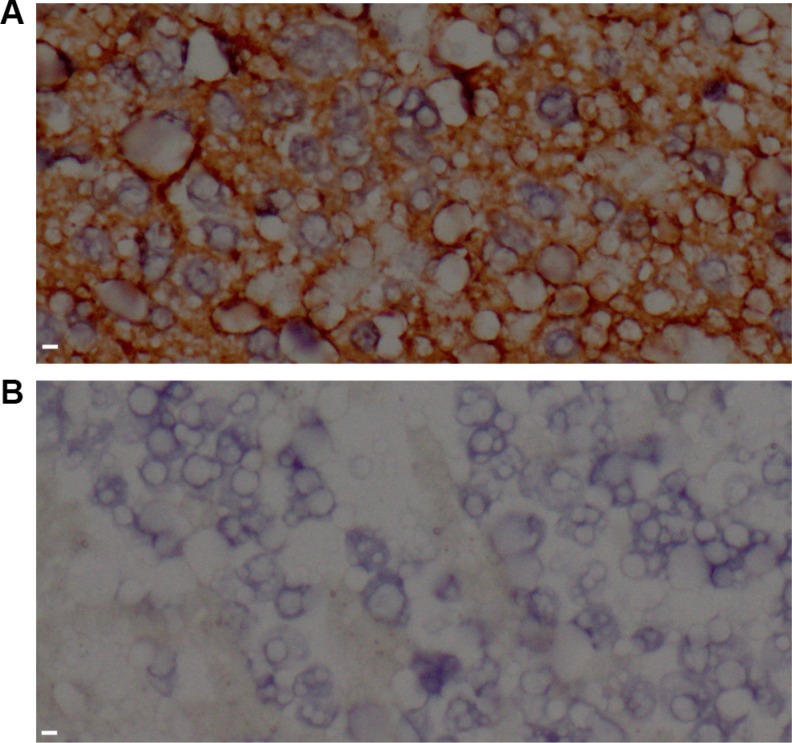
HLA-E expression in tissue from neuroblastoma patients (**A**) Tumor tissue stained positive for HLA-E in a patient with stage IV disease. (**B**) Tumor tissue stained negative for HLA-E in a patient with stage II disease. All images were taken at 400 × magnification. Scale bar is 10 μm.

Eighty-four cases of NB were assessed for HLA-E expression. Overall, 70.2% (59/84) of the tumors stained positive for HLA-E (Figure 1A). HLA-E expression was significantly associated with disease stage. The percentage of HLA-E-positive tumors in patients with stage II, III, and IV disease was 16.7%, 44.4%, and 80.0%, respectively (*P* < 0.01). Thirty-two of the children underwent N-MYC oncogene testing; 10 were positive for N-MYC amplification. The HLA-E expression rates were 90.0% and 50.0% respectively in patients with or without N-MYC gene amplification (*P* < 0.05).

### HLA-E expressed in NB cells inhibited NK-cell cytotoxicity and stimulated cytokine release

**Figure 2:**
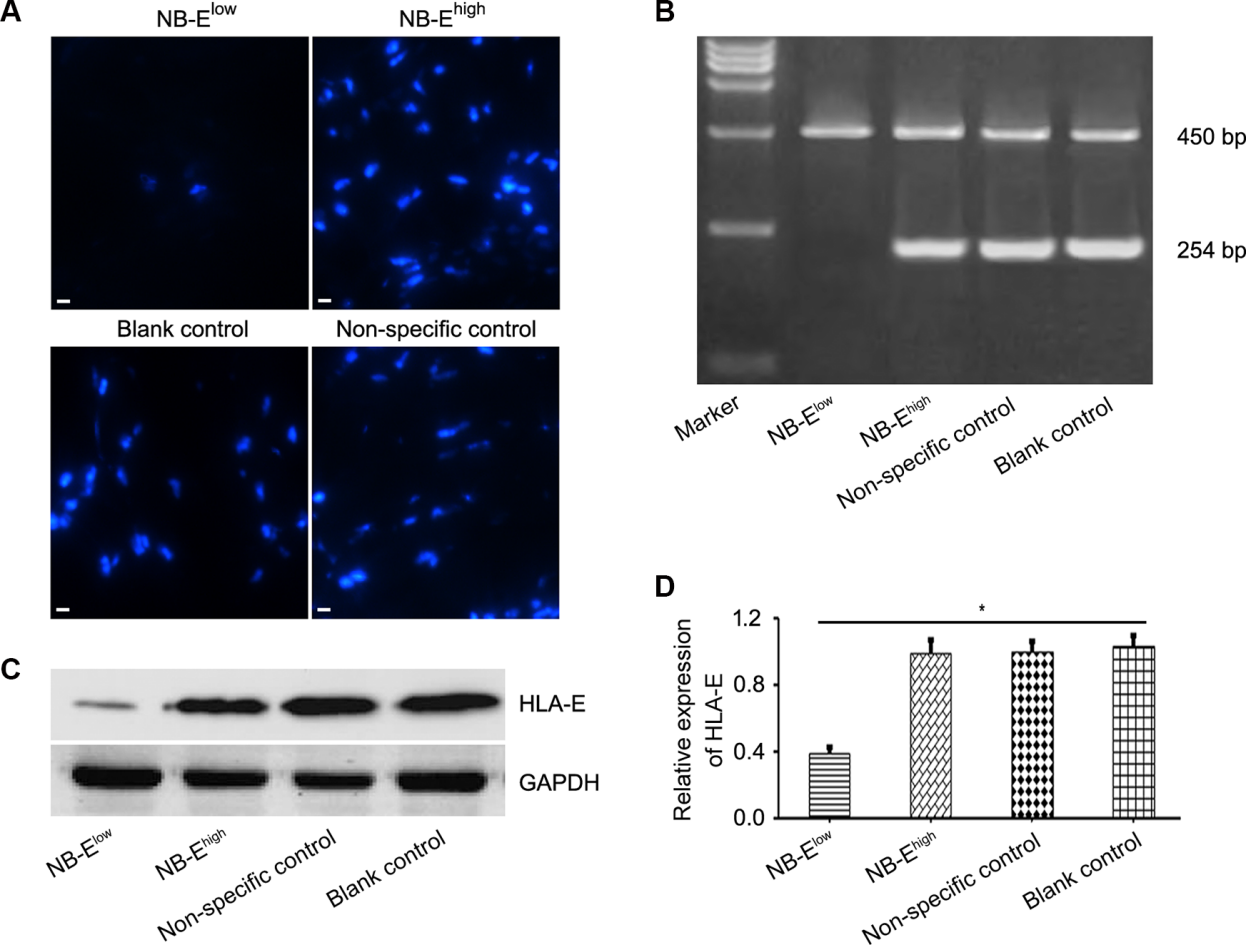
HLA-E expression in neuroblastoma cells (**A**) Immunofluorescence microscopy of NB cells with HLA-E expression in the NB-E^low^, NB-E^high^, and control groups after 48 h of siRNA transfection (× 200). (**B**–**C**) Real-time quantitative PCR and Western blot confirmed that HLA-E was down-regulated in NB-E^low^ cells. (**D**) Relative expression of HLA-E, expressed as IOD^HLA-E^/IOD^GAPDH^, was lower in the NB-E^low^ group than in the NB-E^high^ and control groups. GADPH: endogenous control; IOD: average optical density. The data are representative of five independent experiments. Scale bar is 10 μm. *P < 0.01, two-tailed *t* test.

Cells from the bone marrow of one patient with high HLA-E expression in the tumor tissue and confirmed metastatic disease in the bone marrow were cultured to produce a cell line named NB-E^high^ in this study. NB-E^high^ cells stably expressed high levels of HLA-E (Figure 2). HLA-E expression in NB-E^high^ cells was down-regulated by siRNA transfection, creating NB-E^low^ cells. The blank control, non-specific control, and NB-E^high^ groups expressed HLA-E at high levels at 48 hours, whereas most NB-E^low^ cells expressed little or no HLA-E expression. Western blot analyses showed that NB-E^low^ cells expressed significantly less HLA-E protein than NB-E^high^ and control cells (Figure 2).

**Figure 3:**
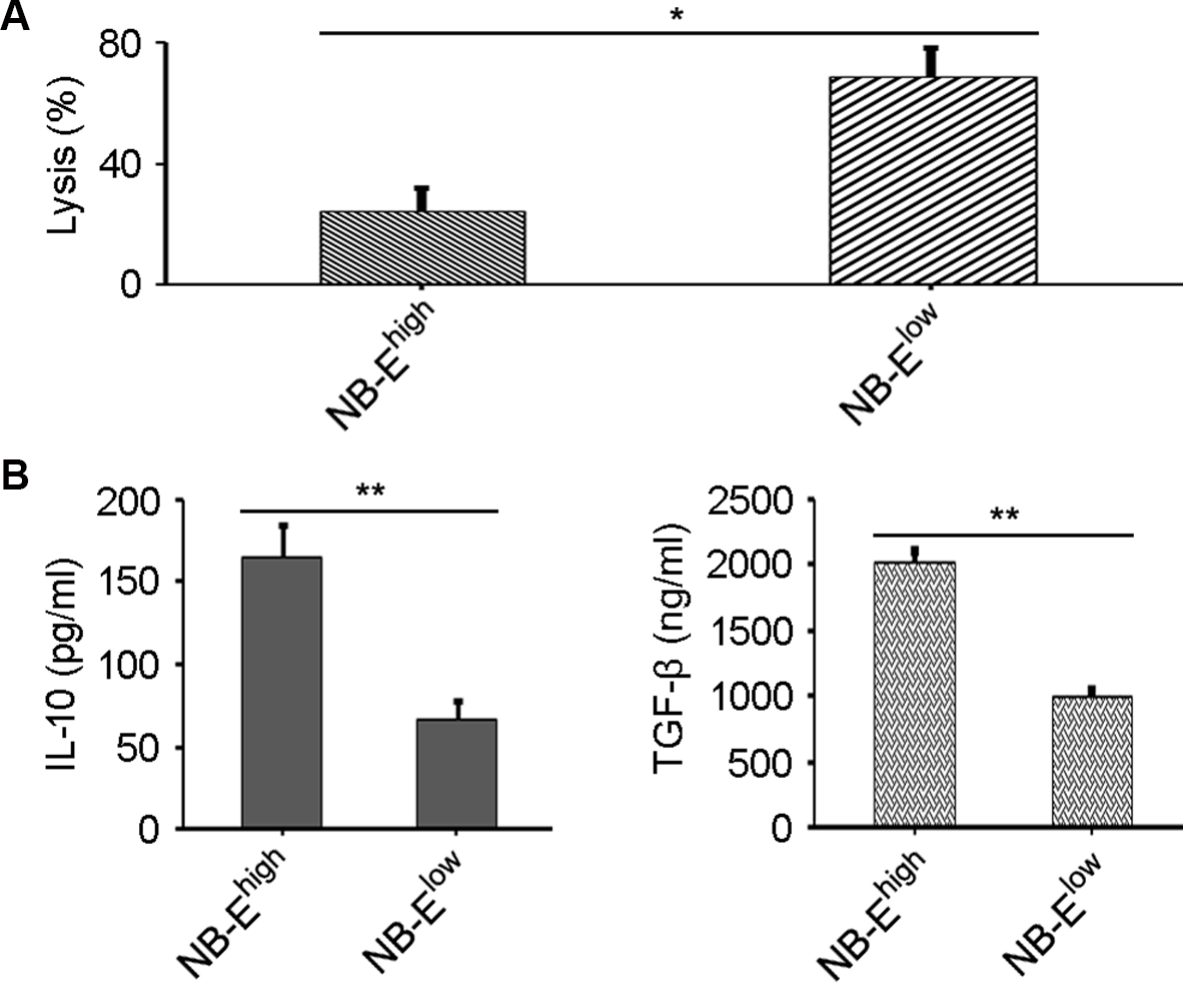
Effect of HLA-E expression on NK-cell cytotoxicity and cytokine release (**A**) Lysis percentage of target cells in the NB-E^low^ and NB-E^high^ groups. The data are representative of five independent experiments. *P < 0.01, two-tailed *t* test. (**B**) Higher IL-10 and TGF-β levels are secreted by NK cells when inhibited by tumor cells in the NB-E^high^ group compared to the NB-E^low^ group. The data are representative of five independent experiments. **P < 0.001, two-tailed *t* test.

NK-cell cytotoxicity was significantly enhanced in the NB-E^low^ group compared to the NB-E^high^ group (Figure 3A). Down-regulation of HLA-E expression in NB cells enhanced NK-cell cytotoxicity toward NB cells. Supernatant was collected after NB cells were co-cultured with NK cells. Sandwich ELISA was used to measure IL-10 and TGF-β in the supernatant. IL-10 and TGF-β levels were higher in the NB-E^high^ group than in the NB-E^low^ group (Figure 3B).

### HLA-E activated the migration and invasion of NB cells *in vitro*

**Figure 4:**
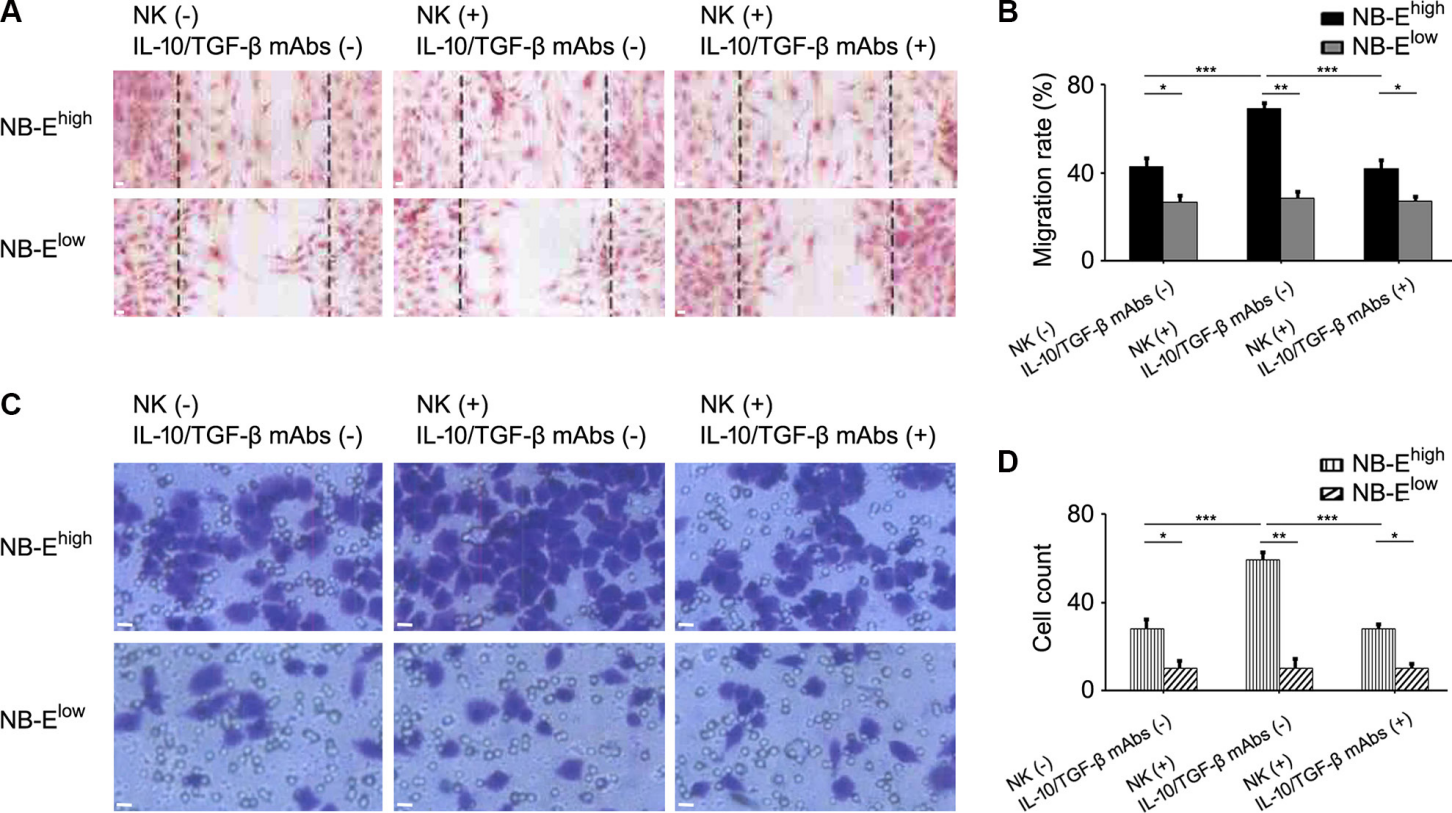
Effect of HLA-E expression on the migration and invasion of NB cells (**A**) Representative migration of NB cells with or without NK/NB supernatant and with or without anti-IL-10/TGF-β mAbs. (**B**) Migration rates of NB cells in the NB-E^low^ and NB-E^high^ groups with or without NK/NB supernatant and with or without anti-IL-10/TGF-β mAbs. The data are representative of five independent experiments. *P < 0.05, ** *P* < 0.001, *** *P* < 0.01. (**C**) Representative invasion of NB cells with or without NK/NB supernatant and with or without anti-IL-10/TGF-β mAbs. (**D**) Invasion rates of NB cells in the NB-E^low^ and NB-E^high^ groups with or without NK/NB supernatant and with or without anti-IL-10/TGF-β mAbs. The data are representative of five independent experiments. Scale bar is 10 μm. *P < 0.05, ** *P* < 0.001, *** *P* < 0.01.

To study the impact of HLA-E expression on the migration and invasion of NB cells *in vitro*, the scratch-wound assay and Transwell invasion assay were adopted. In the scratch-wound assay, the width of the scratch was smaller in the NB-E^high^ group than in the NB-E^low^ group (Figure 4A–4B). The Transwell invasion assay showed more film-crossing cells in the NB-E^high^ group than in the NB-E^low^ group (Figure 4C–4D). When NB-E^high^ cells and NB-E^low^ cells were cultured with NB/NK supernatant, the rate of migration and invasion in the NB-E^high^ group increased, but no significant change was observed in the NB-E^low^ group (Figure 4). When anti-IL-10 and anti-TGF-β mAbs were added to the NB-E^high^ cells cultured with NB/NK supernatant, the width of the scratch and the number of film-crossing cells were similar to those of the NB-E^high^ group without treatment (Figure 4).

### HLA-E promoted NB growth and stimulated cytokine release *in vivo*

**Figure 5:**
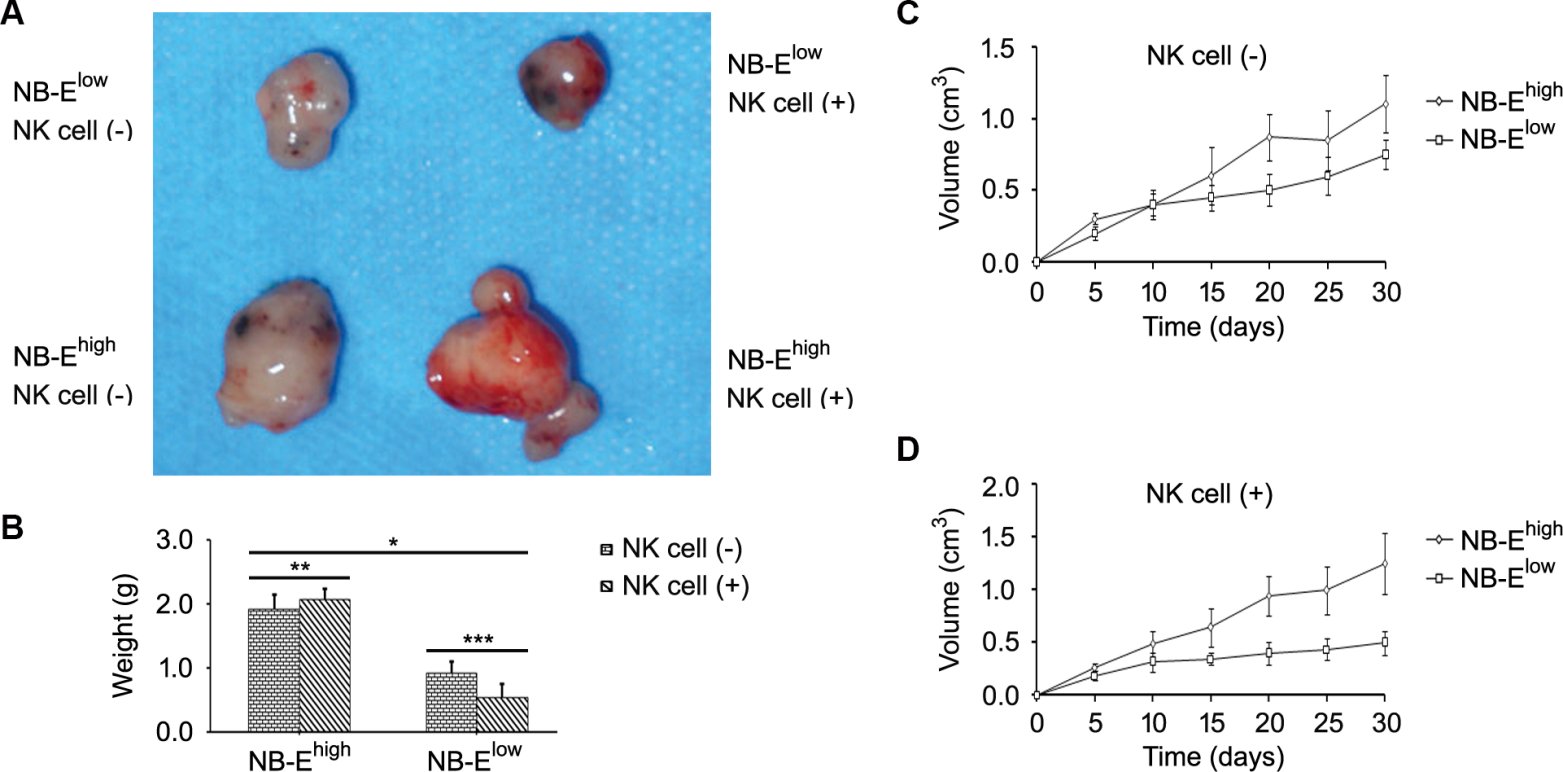
Effect of HLA-E expression on tumor growth (**A**) Representative sizes of the neuroblastoma graft in nude mice. (**B**–**D**) Tumor weight and tumor volume in the NB-E^high^ group and NB-E^low^ group. NK cell infusion decreased tumor weight and volume in the NB-E^low^ group but had no effect on tumor weight and volume in the NB-E^high^ group. *P < 0.01, **P > 0.05, ***P < 0.05, two-tailed *t* test.

**Figure 6:**
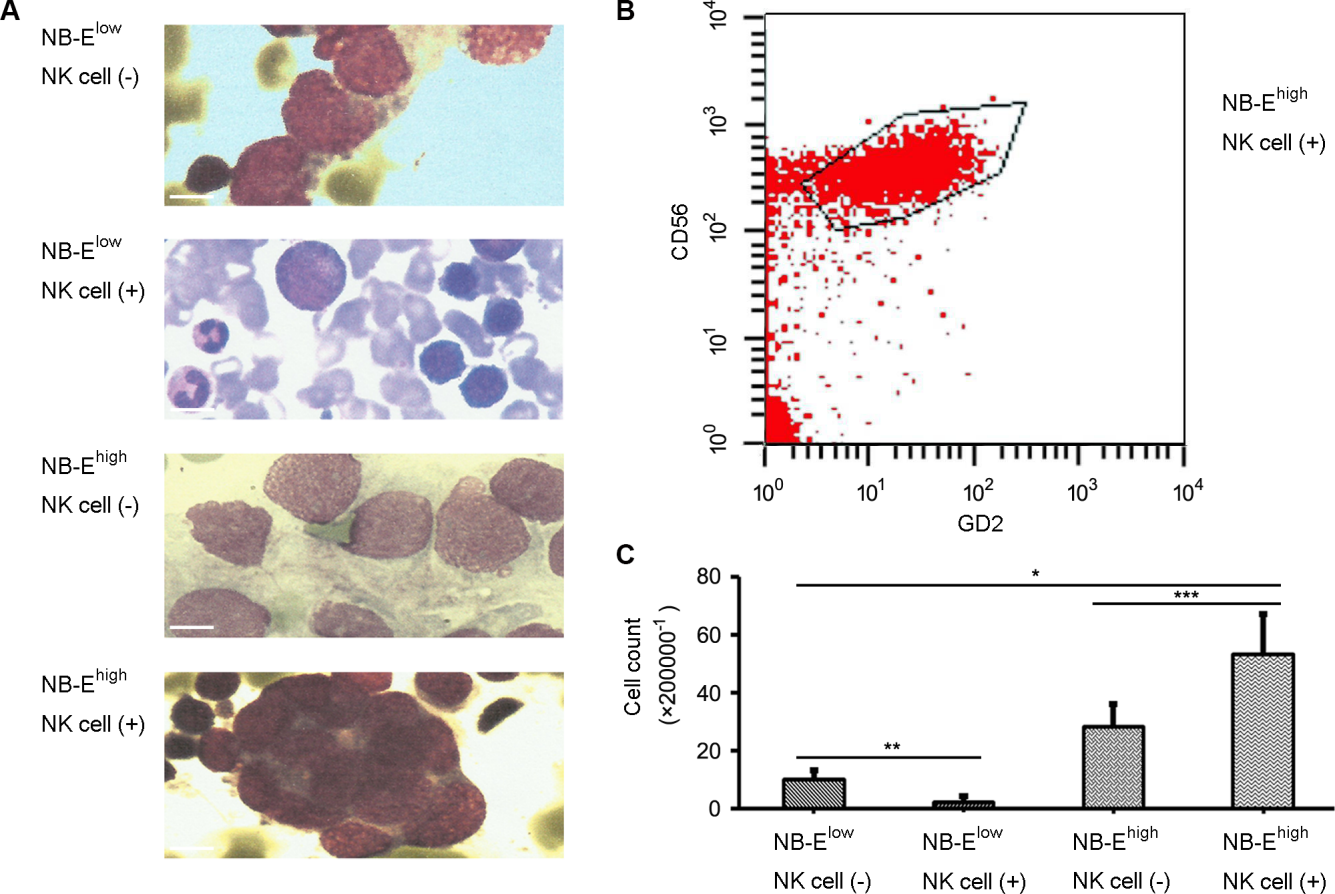
Metastatic neuroblastoma in bone marrow Morphology (**A**) and flow cytology (**B**–**C**) examination confirmed that the number of metastatic cells in the bone marrow was greater in the NB-E^high^ group than in the NB-E^low^ group (*P* < 0.01). NK cell infusion decreased the number of metastatic cells in the bone marrow of mice grafted with NB-E^low^ cells, but increased the number of metastatic cells in the bone marrow of mice grafted with NB-E^high^ cells (*P* < 0.05). Scale bar is 10 μm.

To determine the role of HLA-E expressed in NB cells in tumor growth and cytokine release *in vivo*, a tumor model was establish in nude mice. On day 30 of the growth of the grafted tumor, the NB-E^high^ group had a heavier tumor weight (Figure 5A–5B) and larger tumor volume (Figure 5C–5D) than the NB-E^low^ group. The number of metastatic cells in the bone marrow was greater in the NB-E^high^ group than in the NB-E^low^ group (Figure 6). The expression levels of IL-10 and TGF-β in plasma and tumor tissue were higher in the NB-E^high^ group than in the NB-E^low^ group (Table 1).

**Table 1:** HLA-E-induced expression of IL-10 and TGF-β *in vivo*

Treatment	Group	Peripheral blood			Tumor tissue
		IL-10 (pg/ml)	TGF-β (ng/ml)	IL-10	TGF-β
No NK infusion	NB-E^high^^*^	93.64 ± 15.06	18.9 ± 5.1	++	++
	NB-E^low^	56.07 ± 14.58	12.5 ± 3.5	+	+
NK infusion	NB-E^high^^**^	240.42 ± 23.71	49.3 ± 5.9	+++	+++
	NB-E^low^^***^	63.84 ± 17.13	14.9 ± 4.1	+	+

* all *P* < 0.05 vs. NB-E^low^,

** all *P* < 0.001 vs. NB-E^high^ without NK infusion,

*** all *P* > 0.05 vs. NB-E^low^ without NK infusion.

+ indicates < 10% of positive cells; ++ indicates 10–50% of positive cells; and +++ indicates >50% of positive cells.

### NK cell infusion could not inhibit tumor growth in NB with high HLA-E expression

The effect of NK cell infusion on tumor growth in NB was tested *in vivo*. NK cell infusion significantly increased the expression of IL-10 and TGF-β in plasma and tumor tissue in the NB-E^high^ group, but not in the NB-E^low^ group (Table 1). In mice grafted with NB-E^low^ cells, NK cell infusion decreased the tumor volume, tumor weight and number of metastatic cells in the bone marrow (Figure 5 and Figure 6). However, in mice grafted with NB-E^high^ cells, NK cell infusion had no effect on the local tumor weight (Figure 5A–5B) and tumor volume (Figure 5C–5D), though the number of metastatic cells in the bone marrow was increased (Figure 6).

## DISCUSSION

HLA-E expression is elevated in a number of tumors. Preclinical studies have suggested that tumor cells with high HLA-E expression inhibit NK-cell or CTL cytotoxicity, but blocking the binding between HLA-E and CD94/NKG2A receptor restores NK-cell and CTL cytotoxicity [13]. Similarly, clinical studies have shown that patients with colorectal cancer [14], breast cancer [15], or glioblastoma [16] and high HLA-E expression in the tumor tissue have poorer prognoses. However, contradictory evidence indicates that patients who have cervical [17] or renal carcinoma [18] and high HLA-E expression have good prognoses. Even in the same cancer type, such as colorectal cancer, there are contradictory opinions [19, 14]. Therefore, the effect of HLA-E on tumor prognosis is controversial.

A recent study showed that NB patients with high serum soluble HLA-E (sHLA-E) have a good prognosis. However, that study did not determine whether sHLA-E is released by tumor cells or the immune cells involved in anti-tumor immunity [20]. Our study seemed to have the opposite result. We demonstrated that HLA-E was expressed in the tumor tissues of 70.2% of the NB patients. This high expression of HLA-E was associated with tumor stage and N-myc gene status, which definitively correlated with prognosis. This finding suggested a role of HLA-E in NB growth.

To determine the effect of HLA-E on the cytolytic activity of NK cells, the interaction between NK cells and NB cells with high or low HLA-E expression was observed. siRNA was used to down-regulate HLA-E expression in the NB-E^high^ cells obtained by primary culture to produce the NB-E^low^ cells as confirmed by immunofluorescence assay, Western blot, and quantitative PCR. The cytotoxicity assay showed that NB-E^high^ cells significantly inhibited the cytotoxicity of NK cells towards tumor cells *in vitro*. When HLA-E expression was down-regulated, the cytotoxicity of NK cells was restored as reported in a previous study [3].

HLA-E has been shown to be highly expressed in hepatic cells infected with the hepatitis C virus, inhibit the activity of NK cells, and stimulate NK cells to secrete massive amounts of IL-10 and TGF-β by triggering the NKG2A receptor, modifying the functions of DC cells and CD4^+^ T cells [10]. Similarly, in this study, high IL-10 and TGF-β levels were detected in the supernatant from NK cells co-cultured with NB-E^high^ cells, but not NB-E^low^ cells. This indicates that the release of these cytokines correlated with high HLA-E expression. Recently, TGF-β was found to be related to a pathway important for promoting NB invasion [21] and may be a potential treatment target in NB [22]. IL-10 has also been shown to promote tumor growth via various pathways [23, 12].

Both the scratch-wound assay and Transwell invasion assay showed that NB-E^high^ cells were more capable of migration and invasion than NB-E^low^ cells. The supernatant taken after the co-culture of NB-E^high^ cells and NK cells enhanced the ability of NB-E^high^ cells to migrate and invade tissue. This effect was reversed by the anti-IL-10/TGF-β mAbs, which confirmed that the HLA-E-mediated release of IL-10 and TGF-β promotes tumor infiltration.

In the xenograft experiment in mice, tumors with high HLA-E expression grew rapidly and expressed high levels of IL-10 and TGF-β in plasma and tumor tissue and were associated with metastatic disease in bone marrow. The underlying mechanism of HLA-E-mediated promotion of tumor progression was studied recently at the molecular level [24]. Moreover, we observed that NK cell infusion had an inhibitory effect on NB-E^low^ tumor growth in mice but no effect on local NB-E^high^ tumor growth. Increased IL-10 and TGF-β levels were detected in plasma and tumor tissue from NB-E^high^ mice, as well as the increased metastatic diseases in the bone marrow. Previous studies had proved tumor cell could avoid NK cell attack by direct and indirect mechanismins. Direct mechanisms consisted of shedding soluble ligands for NK cell-activating receptors, upregulation of HLA molecules and release of inhibitory cytokines. Indirect mechanisms consisted of activation of inhibitory regulatory T cells, DC killing, and phagocyte-derived inhibitory cytokines [25]. We speculated HLA-E induced expression of IL-10 and TGF-β by NK cells via these indirect mechanisms in the tumor-NK cell interaction *in vivo*.

Some preclinical studies have shown that NK cell infusion has good efficacy as an immunotherapy for NB [26]. However, NK cells express a complicated range of receptors, such as inhibitory and activating receptors, and their binding to different ligands may affect the efficacy of immunotherapy in NB [27]. Due to the high-affinity of HLA-E for inhibitory CD94/NKG2A receptor and a negative regulatory feedback mechanism in the expression of inhibitory CD94/NKG2A and activating CD94/NKG2C receptors, HLA-E definitively inhibited NK-cell activity [28]. Thus the effect of tumor HLA-E expression on efficacy must be considered when using NK cells as immunotherapy in NB. The present study proved that NK cell infusion does not inhibit the growth of grafted NB with high HLA-E expression in nude mice.

This study did not investigate another HLA-Ib molecule, HLA-G, which is also highly expressed in a number of tumors, including NB [29]. HLA-G also inhibits NK-cell activity and assists tumors in immune escape. However, HLA-G cannot bind directly to CD94/NKG2A; it must first form a complex with HLA-E via its leading peptide and then activates the CD94/NKG2A receptor to deliver an inhibitory signal to NK cells [30]. A recent study using comparative proteomics analyzed 363 proteins expressed in glioma cells and confirmed that the inhibition of NK-cell cytotoxicity is mediated by the HLA-E expressed in tumor cells [31]. Therefore, HLA-G would not have affected the results of the present study.

In conclusion, our study found that high HLA-E expression in NB inhibits NK-cell cytolysis both *in vitro* and *in vivo*. High HLA-E expression also induces NK cells to secrete IL-10 and TGF-β, which affects the promotion of tumor migration and invasion. Because the down-regulation of HLA-E may inhibit tumor growth, HLA-E may be a new treatment target in NB and is worthy of further investigation.

## MATERIALS AND METHODS

### Patients and specimens

All human samples were donated freely, and informed consent was obtained from all children/guardians. Ethical approval was obtained from the Institutional Review Board of Sun Yat-Sen University, Guangzhou, China. Tumor tissues were collected from 84 children (55 boys, 29 girls; median age 4 years, range 2–10 years) with newly diagnosed NB at Sun Yat-Sen University Cancer Center between June 2004 and June 2014. All tissues were obtained after successful clinical diagnosis. The primary tumor sites were the retroperitoneal region in 52 cases, mediastinum in 18 cases, and others in 14 cases. According to the international NB staging system, 6 cases were stage II, 28 cases stage III, and 50 stage IV disease. Thirty-two of the children underwent N-MYC oncogene testing; 10 were positive for N-MYC amplification. Other risk factors, including international NB pathological classification and DNA ploidy, were unavailable at our center and not analyzed in this retrospective cohort. At the time of tissue collection, all patients with stage III and stage IV disease had received reduction chemotherapy, whereas patients with stage II disease were chemotherapy-naive. Due to the long recruitment period and non-uniformity in the treatment protocol, the relationship between HLA-E expression and treatment outcome was not analyzed.

### Immunohistochemical analysis

Conventional formalin-fixed paraffin-embedded tumor tissue was cut into 4-μm sections and stained using the immunohistochemical streptavidin-peroxidase (SP) method [32]. The anti-HLA-E monoclonal antibody 3H2679 (Santa Cruz, USA) was used. HLA-E expression was evaluated as described previously [5].

### Primary culture

Cells from the bone marrow of one patient with high HLA-E expression in the tumor tissue and confirmed metastatic disease in the bone marrow were obtained at diagnosis. The cells were suspended in Dulbecco's Modified Eagle Medium (DMEM) containing 10% fetal calf serum, 1% non-essential amino acids, and 1% penicillin/streptomycin and distributed into 25 cm^3^ cell culture flasks. Primary cultures were incubated at 37°C with 5% CO_2_ in air. After collecting the tumor cells, high HLA-E expression on the cell surface was confirmed by Western blotting and polymerase chain reaction (PCR). These cells were referred to as NB-E^high^ in this study.

### Cellular siRNA transfection

A siRNA sequence was designed based on the mRNA sequence of HLA-E (NM_005516) [33]. The target region was 525-547: ATCTCCGAGCAAAAGTCAAATGA. The sense primer was 5′-CUCCGAGCAAAAGUCA AAUGA-3′ and the anti-sense primer 5′-AUUUGACUUU UGCUCGGAGAU-3′. NB-E^high^ cells were seeded in 24-well plates (5 × 10^4^ cells/well) and cultured for 18 h before transfection. Transfection was performed using 50 pmol siRNA and 2.5 μl Lipofectamine 2000 at a total volume of 0.5 ml using a previously reported method [34]. The goal was to obtain a cell line with low HLA-E expression, referred to as NB-E^low^. PBS and non-specific siRNA were used to establish a blank control and non-specific control, respectively. The effect of silencing the HLA-E gene was detected by immunofluorescence, Western blotting, and real-time quantitative PCR as reported previously [35–37].

### NK-cell cytotoxicity

A 2 ml venous blood sample was taken from healthy individuals, mixed with lymphocyte separation medium, and centrifuged (2000 r·min^−1^ × 20 min) to obtain peripheral mononuclear cells. The magnetic bead system coated with anti-CD56 monoclonal antibody was used to select NK cells as effector cells. NB cells (i.e., target cells) were added to 96-well plates at a density of 2.5 × 10^4^ cells/well and cultured for 14 hours. The cultured NB cells were combined with the effector cells at an effector/target ratio of 100:1 and cultured for 20 hours. The activity of adherent target cells in the wells was measured using the methyl thiazolyl tetrazolium (MTT) method. The rate of NK-cell cytotoxicity was calculated as reported previously [38].

### Enzyme-linked immunosorbent assay (ELISA)

NB cells at a density of 1 × 10^6^ cells/well were cultured with NK cells at an effector/target ratio of 100:1. The supernatant was collected and tested for IL-10 and TGF-β using an ELISA kit (Phoenix Pharmaceuticals Inc., US). The supernatant from NK cells cultured with NB-E^high^ and NB-E^low^ cells was termed NB/NK supernatant 1 and NB/NK supernatant 2, respectively, and stored for further usage in scratch-wound assay and Transwell invasion assay.

### Scratch-wound assay

The migration capability of NB cells with different HLA-E expression was determined by the scratch-wound assay as previously described [39]. To investigate the impact of IL-10 and TGF-β secreted by NK cells on NB cell migration, NB-E^high^ and NB-E^low^ cells were cultured with NB/NK supernatant 1 and NB/NK supernatant 2, respectively, and the scratch-wound assay repeated. Anti-IL-10 and anti-TGF-β monoclonal antibodies (mAbs) (Santa Cruz, USA) were added (5 μg/ml each) to the NB cells cultured with NB/NK supernatant to determine whether the promotion of NB cell migration by NB/NK supernatant was impaired.

### Transwell invasion assay

The invation capability of NB cells with different HLA-E expression was determined by the Transwell invasion assay as previously described [40]. To investigate the impact of IL-10 and TGF-β secreted by NK cells on NB cell invasion, NB-E^high^ and NB-E^low^ cells were cultured with NB/NK supernatant 1 and NB/NK supernatant 2, respectively, and the scratch-wound assay repeated. Furthermore, anti-IL-10 and anti-TGF-β mAbs (5 μg/ml each) were added to the NB cells cultured with NB/NK supernatant to determine whether the promotion of NB cell invasion by NB/NK supernatant was impaired.

### Animal model

NB cells were inoculated in the right armpits of Balb/c nu/nu nude mice (Laboratory Animal Center, Southern Medical University, Guangzhou, China) at a density of 1 × 10^6^ cells/200 μl to establish the NB model. On day 30 after inoculation, the mice were sacrificed and the tumor tissue obtained and weighed. ELISA was used to measure IL-10 and TGF-β in the peripheral serum from the nude mice. Tumor tissue was obtained and semiquantitative immunohistochemistry performed to measure the expression of IL-10 and TGF-β as described previously [41]. In tumor tissue, reactions were grouped by intensity, quantity, and type of staining (intensity: absent, weak, medium, strong; quantity: no positive tumor cells, < 10% of cells, 10–50% of cells, > 50% of cells).

### NK cell infusion

The NB xenografts in nude mice were established and grouped as described previously. NK cells from healthy volunteers were stimulated overnight with a combination of 10 ng/ml IL-15 and 40 IU/ml IL-2. A total of 5 ×10^5^ activated NK cells were infused in each of the mice via the caudal vein weekly over a total of 4 weeks. On day 30 after inoculation, the mice were sacrificed and the tumor tissue obtained and weighed. ELISA was used to measure the peripheral serum levels of IL-10 and TGF-β in the nude mice. Immunohistochemistry was used to determine the expression profile of IL-10 and TGF-β in the grafted tumor tissue.

### Bone marrow cytology and flow cytology

To quantitatively evaluate the NB metastatic disease in nude mice on day 30, bone marrow, which is one of the most commonly involved sites in NB, was taken from the left femur for examination by morphology and flow cytology. FCM (FACScalibur^TM^, Becton-Dickinson, US) analysis was performed, as previously described [42], on the bone marrow specimens using the antibodies of anti-human ganglioside D2 (GD2) monoclonal antibody, phycoerythrin (PE) labeled anti-neural cell adhesion molecule monoclonal antibody (CD56) and peridinin chlorophyll protein (PerCP) labeled CD45 monoclonal antibody.
